# Pomegranate: A Source of Multifunctional Bioactive Compounds Potentially Beneficial in Alzheimer’s Disease

**DOI:** 10.3390/ph16071036

**Published:** 2023-07-21

**Authors:** Lidia Ciccone, Susanna Nencetti, Armando Rossello, Elisabetta Orlandini

**Affiliations:** 1 Department of Pharmacy, University of Pisa, Via Bonanno 6, 56126 Pisa, Italy; susanna.nencetti@unipi.it (S.N.); armando.rossello@unipi.it (A.R.); 2 Research Center “E. Piaggio”, University of Pisa, 56122 Pisa, Italy; elisabetta.orlandini@unipi.it; 3 Department of Earth Sciences, University of Pisa, Via Santa Maria 53, 56126 Pisa, Italy

**Keywords:** Alzheimer’s disease, AD, pomegranate fruit, nutraceuticals, antioxidants, multifunctional compounds, neuroprotection, ellagic acid, gut-microbial, urolithins

## Abstract

Pomegranate fruit (PF) is a fruit rich in nutraceuticals. Nonedible parts of the fruit, especially peels, contain high amounts of bioactive components that have been largely used in traditional medicine, such as the Chinese, Unani, and Ayurvedic ones, for treating several diseases. Polyphenols such as anthocyanins, tannins, flavonoids, phenolic acids, and lignans are the major bioactive molecules present in PF. Therefore, PF is considered a source of natural multifunctional agents that exert simultaneously antioxidant, anti-inflammatory, antitumor, antidiabetic, cardiovascular, and neuroprotective activities. Recently, several studies have reported that the nutraceuticals contained in PF (seed, peel, and juice) have a potential beneficial role in Alzheimer’s disease (AD). Research suggests that the neuroprotective effect of PF is mostly due to its potent antioxidant and anti-inflammatory activities which contribute to attenuate the neuroinflammation associated with AD. Despite the numerous works conducted on PF, to date the mechanism by which PF acts in combatting AD is not completely known. Here, we summarize all the recent findings (in vitro and in vivo studies) related to the positive effects that PF and its bioactive components can have in the neurodegeneration processes occurring during AD. Moreover, considering the high biotransformation characteristics of the nutraceuticals present in PF, we propose to consider the chemical structure of its active metabolites as a source of inspiration to design new molecules with the same beneficial effects but less prone to be affected by the metabolic degradation process.

## 1. Introduction

Neurodegenerative diseases are multifactorial disorders characterized by common pathological processes that lead to irreversible loss of neuronal functions. The neurological damage is the result of several complications related to protein misfolding and aggregation, altered levels of metals and neurotransmitter concentrations, oxidative stress (OS), and neuroinflammation [1,2].

Despite the huge effort to understand the molecular mechanisms behind the pathogenesis of neurodegenerative disorders, they remain elusive. In the following paragraphs, we give an overview of the different mechanisms involved in Alzheimer’s disease (AD) onset and progression for a better understanding of the beneficial effects that pomegranate diet intake could have in AD.

In AD, the widespread theory about pathogenesis onset is the amyloid cascade hypothesis [3] which suggests that the accumulation of β-amyloid (Aβ) peptides leads to toxic aggregates favoring Aβ plaque deposition in the brain. This happens when Aβ production is not balanced with its clearance. Aβ peptides are the products derived from the proteolytic cleavage of the amyloid precursor protein (APP) by β and γ-secretases (amyloidogenic pathway) [4]. In addition to the extracellular deposition of Aβ plaques, the brain tissue from AD patients is characterized by the presence of intracellular neurofibrillary tangles (NFTs) of hyperphosphorylated tau proteins [5].

Tau is a protein predominantly expressed in neurons and glial cells, in the central nervous system (CNS) and, under physiological conditions, is involved in the stabilization and polymerization of microtubules, regulation of axonal transport, and axon growth [6]. In AD, hyperphosphorylation of tau promotes its dissociation from microtubules, decreasing microtubule stability and favoring tau oligomerization and aggregation into toxic NFTs [7,8,9]. For many years, AD disease models have suggested that Aβ trigged a pathophysiological cascade leading to tau misfolding, resulting in neurodegeneration and cognitive decline. However, several pieces of experimental evidence now suggest that there is a synergy between Aβ and tau, and the microglia could be the key intermediate [10]. The Aβ–tau interaction is an example of negative protein–protein cross-interaction [11]. Recently, positive cross-interactions between Aβ and other proteins, such as transthyretin, have been studied as a possible innovative therapeutic approach against AD progression [12,13,14].

Several studies highlighted the important role that neuroinflammation plays in the progression of AD. The inflammation hypothesis for AD is related to the abnormal activation of proinflammatory agents and the chronic neuroinflammatory state characteristic of AD patients [15]. Experimental evidence reports that Aβ can also provoke neuronal damage through activation of microglia, promoting the secretion and the release of neurotoxic and proinflammatory cytokines which cause inflammation [16]. Moreover, microglial activation is a critical point for Aβ clearance from the brain via endocytosis [17]. In this context, therapeutic approaches targeting neuroinflammation and microglia activation are an attractive research area in AD [18].

The cholinergic hypothesis for AD is related to the neurotransmitter alteration found in the brain of patients where acetylcholinesterase (AChE) was found co-localized with Aβ peptide deposits [19]. An abnormal activation of AChE leads to a decrease in acetylcholine (ACh) neuronal levels with consequently slowing down of learning and memory processes. Currently, three of the drugs approved for the treatment of AD are AChE inhibitors (donepezil, galantamine, and rivastigmine), one is an uncompetitive antagonist of *N*-Methyl-D-aspartate receptors (NMDA) (memantine), and the Food and Drugs Administration approved two monoclonal antibodies against Aβ (aducanumab and lecanemab) [20,21,22,23].

Although the abnormal accumulation of aggregate proteins is the main molecular signature of neurodegenerative diseases, other features such OS, nitrosative stress (NOS), and mitochondrial dysfunction have a detrimental role in the pathogenesis of AD [24,25]. Reactive oxygen species (ROS) can provoke nucleic acid breakage, polysaccharide depolymerization, lipid peroxidation, and other dramatic effects that cause damage to neurons and cell death [26,27]. Experimental evidence highlights that there is a strict correlation between the aggregates of amyloid proteins, mitochondrial damage and dysfunction, and ROS production [28,29].

Metal ions play an essential role during the physiological process, indeed most of the time proteins need at least one metal ion to function. In contrast, dyshomeostasis of metals is responsible for various pathological complications previously mentioned such as OS, mitochondrial dysfunctions, and amyloid-forming proteins [30,31]. Post-mortem investigation of Aβ plaques in the brains of AD patients, showed higher accumulations of Cu, Fe, and Zn ions, compared to the normal levels detected in healthy brains, confirming that physiological metals play a key role in AD [32,33].

To address a plethora of factors which characterize the multifactorial pathology of AD, scientists have focused their attention on a multi-target approach [34]. In this context, natural compounds gained attention for their intrinsic multifunctional nature, and semisynthetic and fully synthetic molecules inspired by natural compounds have been largely studied for their potential effects against AD and neurodegenerative diseases [35,36,37,38,39,40,41].

Pomegranate fruit (*Punica granatum* L.) (PF) has been recognized as a fruit rich in bioactive molecules. It has been extensively used in traditional medicine, such as the Chinese, Unani, and Ayurvedic ones, for treating several diseases. PF is one of the fruits that contains the highest amount of polyphenols such as anthocyanins, tannins, flavonoids, phenolic acids, and lignans [42]. The nutraceuticals are contained both in the edible part of the fruit (pulp and seeds) and in the peel, leaves, flowers, and hull, the processing by-products of the plant. The flower of its plant was used for its astringent and hemostatic properties, in injuries and in the treatment of diabetes [43,44].

**Figure 1:**
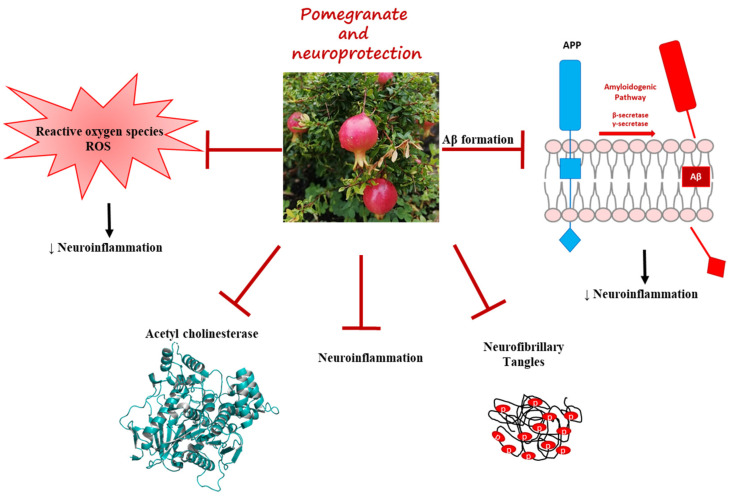
Graphical representation of the beneficial effects that pomegranate can have in the neurodegeneration process. Crystal structure of acetylcholinesterase (PDB 5HFA) made by PyMol slightly modifying the script previously used [45].

Recently, several studies report that the bioactive molecules contained in pomegranate seed (PS), pomegranate peel (PP), and pomegranate juice (PJ) have a potential beneficial role in AD against the formation of ROS, reducing neuroinflammation, inhibiting AChE, and decreasing the Aβ plaques and NFTs (Figure 1).

In this review, we report the most recent studies conducted on pomegranate with the aim to give an overview about the potential neuroprotective role that PF can have against AD.

## 2. Pomegranate Fruit (PF)

Nutraceuticals involve several product categories derived from foods of vegetal and animal origin. The term nutraceutical is used for bioactive natural products that have a “possible beneficial role” for health [46,47]. In the last few years, PF and its extracts have been largely studied for their coadjutant activities in preventing or treating numerous chronic pathologies such as cancer, diabetes, atherosclerosis, cardiovascular, and neurodegenerative diseases [48,49,50,51,52,53]. The pomegranate peel (PP) and its extract (PPE) have nutraceutical properties because they are rich in antioxidant, antimicrobial, antiatherogenic, and antimutagenic compounds [54,55]. Folk medicine traditionally recommends the use of PP dried for the treatment of various disorders, such as colitis, headache, aphthae, diarrhea, dysentery, and ulcers [56].

The peel represents approximately 50% of the pomegranate weight and it consists of the outer skin, mesocarp, and divider membrane. PP is characterized by high-molecular-weight phenolic molecules such as ellagitannins, proanthocyanidins, complexes with polysaccharides, and flavonoids [42]. Among the tannins, the major constituents in the PP are the antioxidant ellagitannins that are easily hydrolyzed in ellagic acid (EA) by gut microflora. Punicalagin isomers, ellaginnis derivatives, are the major components extracted by PP; they are also present in the roots even if in lower amounts than in PP [57]. The pomegranate juice (PJ) contains a great quantity of anthocyanins, flavonoids, and hydroxyl benzoic acids [58,59]. Pomegranate seeds (PS) are rich in unsaturated–polyunsaturated fatty acids, vitamins, sugar, polysaccharides, polyphenols, and minerals. The PS extract and oil are characterized by a great amount of fatty acids, linoleic acid, gallic acid, and EA [60]. The pomegranate leaves (PL) are rich with hydrolysable tannins such as granatins A and B, while there is only a trace of punicalagins and punicalins. Pomegranate flowers are characterized by several phytochemicals such as EA and two of its oxidized derivatives (pomegranatate and phyllanthusiin E, punnicatannins A and B, isocorilangin, etc.) [43].

Recently, nutraceuticals derived from PF have been largely studied [42,44,56,61] for their multifunctional nature. In the next paragraphs, we report the recent finding about the potential positive effect that pomegranate and its nutraceuticals have in AD.

### 2.1. Pomegranate Peel Extract and Its Bioactive Compounds in AD

**Figure 2:**
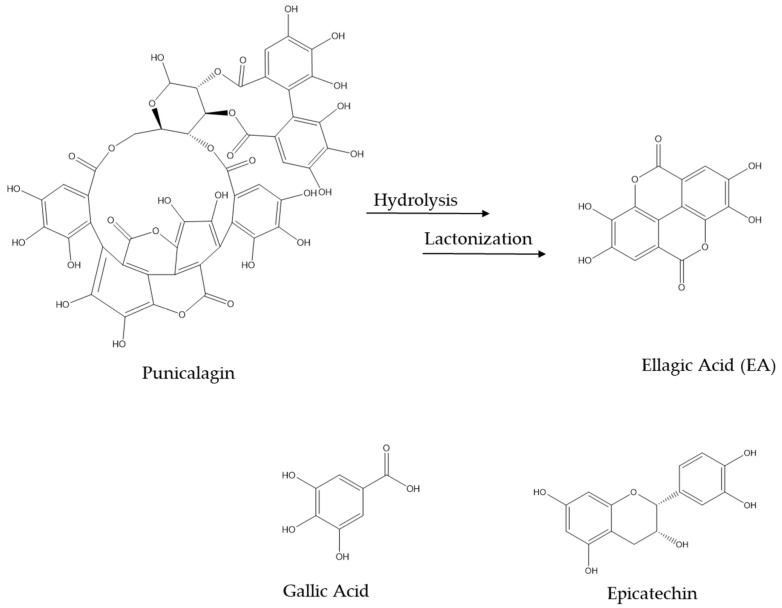
Chemical structures of the major components present in PPE. SMILES from PubChem.

Studies display that PP possesses higher levels of bioactive compounds compared to the edible pulp of the fruit [62,63,64,65]. Experimental data suggest that extracts from PP (PPE) are characterized by compounds, such as EA and punicalagin (Figure 2), able to inhibit β-site APP Cleaving Enzyme 1 (BACE1), IC_50_ 3.9 × 10^−5^ M and 4.1 × 10^−7^ M, respectively, contrasting the Aβ production [66]. Moreover, in order to investigate the enzyme specificity, EA and punicalagin were also tested on tumor necrosis factor alpha converting (TACE) and other serine proteases (chymotrypsin, trypsin, and elastase). Interestingly, up to 40 μM, EA and punicalagin inhibited more than 80% of BACE1 while no effect was detected against the other tested enzyme [66]. Thus, for the first time it was proved that EA and punicalagin were BACE1 inhibitors, exercising their action directly on the enzyme.

An in vivo study reported that mice of AD model (male C57BI/6 Aβ infused) treated with PPE (800 mg/kg/day) showed less failure to find the escape box in the spatial memory Barnes maze test [67]. Before administration, PPE was characterized and a high amount of phenolic compounds (21.25 ± 0.16 mg of gallic acid/g) and flavonoids (7.60 ± 0.12 mg of quercetin/g), strong antioxidants, were found. Moreover, punicalagin was present at a concentration of 13.74 mg/g in PEE. In the same study, the senile plaques quantification was investigated in six hemispheres from different mice. Interestingly, the mice infused with Aβ and treated with PPE showed a remarkable reduction in the number of plaques compared to the mice infused with Aβ without PPE administration. This result can be attributed to EA and punicalagin (Figure 2); two main components of PPE that, as previously mentioned, are able to inhibit β and γ-secretase inhibiting Aβ plaque formation [66,67]. These animals were also studied to determine the brain-derived neurotrophic factor (BDNF) density. BDNF plays a key role in maintaining synaptic plasticity. The Aβ mice treated with PEE showed a relevant increase in the neurotrophin compared to the control and Aβ groups [67], suggesting that PPE has a neuroprotective effect. Moreover, in vivo and in vitro studies reported that PPE had an inhibition action against the AChE. Aβ mice treated with PPE showed remarkable AChE activity in the cortex and hippocampus compared to Aβ groups, decreasing the cholinergic deficits [67]. Finally, in agreement with the literature [55,68,69], PPE in the Aβ mice group showed good antioxidant activity both reducing the lipid peroxidation in thiobarbituric acid reactive substances (TBARS) (liver homogenates) and reducing the expression of the cytokine, such as tumor necrosis factor (TNF)-α [67]. In addition, it is interesting to highlight that in Aβ mice treated with PPE, no hepatic lesions were observed.

Merging together all the data collected in this study, it appears clear that PPE simultaneously reduces the accumulation of Aβ plaques, the activity of AChE, the lipid peroxidation, and the expression of the inflammatory cytokine TNF-α, while increasing BDNF in the AD mice model. Therefore, PPE, thanks to its nutraceuticals, acts as a neuroprotective agent triggering several mechanisms to combat AD progression in Aβ mice.

Investigations have been conducted to understand if the neuroprotective effects are related only with the single constituent EA, one of the abundant components of the PPE.

AD rat models were prepared by administering AlCl_3_ orally; one group was treated with PPE (POMELLA^TM^, Verdure Sciences, Noblesville, IN, USA, 50 mg/kg), another with EA (50 mg/kg) for twenty-eight days. In the behavioral tests of radial arm maze (RTM), the rats belonging to both groups showed a relevant reduction of errors; however, the animals treated with PPE displayed less errors than those treated with EA [70]. A similar tendency was found when investigating the antioxidant properties. The quantification of catalase and glutathione, two biomarkers to evaluate the antioxidant activity, in the hippocampus homogenate showed that they were drastically reduced in AD rats treated with PPE, while a moderate decrease was found in AD + EA rat models. The same result was also found in the TBARS test and in the total antioxidant capacity (TAC) assay. The histopathological analysis confirms that in the AD + PPE rats, neurofibrillary tangles and senile plaques (the two main hallmarks of AD) were reduced compared to the control and AD + EA animals [70].

In this study, monitoring the levels of catalase, glutathione, TAC, and malondialdehyde (MDA), it was proved that OS is an important factor in the neurodegenerative AD mice model. Therefore, the administration of antioxidants mitigates the AD side effects, in particular, PPE was more potent than the single molecule of EA.

The results reported in the literature, both in vivo and in vitro studies, agree with the hypothesis that PPE has neuroprotective actions. The higher effect of PPE compared to a single component could be attributed to the copious amount of bioactive and multifunctional molecules contained in PPE that probably act simultaneously against several neurodegenerative pathways.

### 2.2. Pomegranate Juice and Extracts against AD

Pomegranate is usually consumed as whole fruit, juice, or botanical dietary extracts. In recent years, pomegranate has become popular for its numerous benefits related to human health; thus, numerous different pomegranate-based products appeared on the market such as powdered capsules and tablets, tea, jam, wine, jelly, and spices [71].

**Figure 3:**
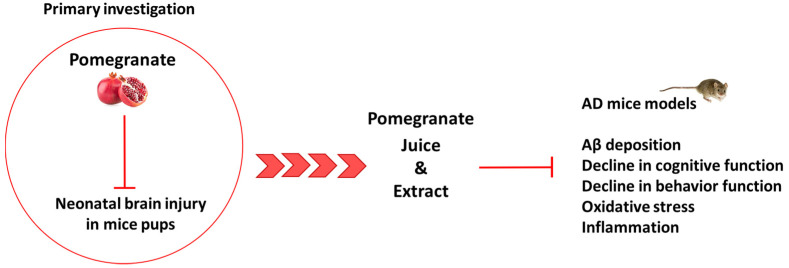
Workflow history of the potential neuroprotective effects of PJ and pomegranate extract (PE) in AD animal models.

Experimental evidence suggests that feeding disorders, like malnutrition and obesity, have an impact on AD [72,73,74], whereas a diet based on foods rich in vitamins and polyphenols seems to be able to prevent and reduce the AD onset [75]. Regarding the human studies, more data needs to be collected in order to better understand the positive relationship between polyphenol and AD [76], while several animal model studies support this hypothesis. This paragraph summarizes the animal model studies focused on the potential neuroprotective effects of PJ and pomegranate extract (PE) on AD (Figure 3).

#### 2.2.1. Pomegranate Protects Pups’ Mice from Brain Injury

The first experimental evidence about the neuroprotective action of PJ was found in the animal model of neonatal hypoxic–ischemic (H–I) brain injury. When the maternal mice’s diet was enriched with PJ, pre- and post-injury, pups were protected against neonatal brain injury [77]. Interestingly, EA, a component of PJ, was found in plasma collected from pups that were exposed to maternal PJ supplementation but not in mice from the control group, suggesting that PJ components can cross the mouse placenta and move from maternal to pup serum [77]. In order to verify that the protective role of PJ is due to the polyphenol contained in the juice, the effect of the pomegranate polyphenol extract was studied in the same neonatal H–I brain injury model [78]. The pomegranate polyphenol-enriched extract (PPEE) was prepared using the skin and the aril; the dose of polyphenols was estimated to be more or less the same of that contained in the PJ used in the previous study. During pregnancy and following delivery, mice were treated with sugar water (vehicle) or PPEE in vehicle. Pups of dams that were fed with drinking water added with PPEE showed a relevant decreased H–I-induced caspase-3 activation. This result is in agreement with the hypothesis that the polyphenols of PJ have a neuroprotective role [77,78].

#### 2.2.2. Pomegranate Effects in AD Model

The promising experimental evidence concerning the neuroprotective effect of PF inspired the researchers to investigate if dietary supplementation with PJ would have any effects on the AD mouse model. Transgenic mice APP_sw_/Tg2576 were used as an AD model because they express a form of APP that induces Aβ deposition speeding AD onset [79]. Starting from 6 months of age, APP_sw_ mice were treated with vehicle or PJ in vehicle. PJ was prepared using the Wonderful variety of pomegranates and its composition was determined experimentally (84% water, 14% carbohydrates, 0.48% ash, 0.4% citric acid, 0.1% protein, 0.02 fat, and 1% other, phenolic acids, and flavonoids). The PJ phenolic acid content was composed of 115 ppm EA and 5 ppm gallic acid, while flavonoids included 1880 ppm hydrolysable tannins (gallotannins, ellagitannins, punicalagin) and 369 ppm anthocyanins and their glycosides derivatives (cyaniding, delphinidin, pelargonidin) [79].

At one year of age, APP_sw_ mice were evaluated. The learning and memory ability of APP_sw_ mice administered with PJ showed a generally better profile compare to APP_sw_ mice treated with vehicle using the Morris water maze test (both CUED, visible platform, and SPATIAL, hidden platform) [80]. Moreover, in the hippocampus of PJ-treated APP_sw_ mice, the levels of soluble Aβ_42_ (51%; *p* < 0.004) and Aβ deposition (fibrils 50% *p* < 0.008; and amyloid 53% *p* < 0.03) were strongly reduced with respect to APP_sw_ control mice [79] (Table 1). Additionally, in the hippocampus of mice treated with PJ, the Aβ_1–42_:Aβ_1–40_ ratios were drastically reduced. One hypothesis proposed by the authors was that the reduced amount of Aβ was related to the ability of PJ to inhibit β-secretase. Unfortunately, the analysis of the β-C terminal fragment (β-CTFs) showed that, in the mice treated with PJ, β-secretase was not affected suggesting that the mechanism of action by which PJ acted on Aβ is not related to APP processing or Aβ production [79]. However, the results obtained were in line with other published studies that reported the ability of polyphenols to decrease the levels of Aβ and Aβ deposition in the brain of APP transgenic mice [81]. Interestingly, this was the first study that proved the potential beneficial action of PJ in the AD animal model.

In the following years, other in vivo studies suggested that dietary supplementation with pomegranate can contribute to slow the loss of cognitive and behavioral functions in AD.

The effect of Oman pomegranate was also evaluated in long-term dietary supplementation in APP_sw_/Tg2576. Starting from the age of four months, the diet of a group of APP_sw_ mice was enriched with 4% PE, and the behavioral and functional properties were analyzed after a long period of time (14–18 months). The PE was prepared starting from fresh PF collected in Oman and the total phenolic content was found to be 693.472 ± 0.632 (mg of gallic acid/100 g). The spatial memory and learning ability, psychomotor coordination, and anxiety-related behavior were evaluated using the Morris water maze, rotarod performance, elevated plusmaze, and open field tests. The results obtained clearly showed that mice treated with 4% PE were characterized by relevant attenuation of learning and memory deficits, decreased anxiety, and improved motor coordination [82] (Table 1).

In contrast with this result, in another study, transgenic AD mice (R1.40, aged 24–30 months) fed with PE (at 100 and 200 mg/kg) did not show improvements in cognitive performance. While in accordance with previous published data, in the AD mice treated with PE, a different ratio and amount of Aβ_1–42_ and Aβ_1–40_ was found to be contributing to inhibit AD progression [83], Table 1. The authors suggested that the changing of Aβ concentration was due to the action of PE on γ-secretase enzyme activity which appeared modified.

The Aβ plaques and NFTs, two main hallmarks of AD, are strictly connected to chronic inflammation and neuronal dysfunction. Even if the molecular mechanism of AD is not completely known, studies report that Aβ_1–42_ and NFTs can trigger the neuroinflammatory process through the activation of microglia, astrocytes, and the induction of proinflammatory cytokines [89,90]. In this context, the proinflammatory molecules such as nuclear factor of activated T cells (NFAT), interleukin (IL)-1β, IL-6, and tumor necrosis factor-α (TNF-α) have been reported to take part in neuritic plaque formation in AD [91,92,93]. The NFAT is expressed in microglia where it plays a role in regulating proinflammatory responses [94,95]; therefore, some authors hypothesized that the discovery of molecules capable of acting as NFAT inhibitors could be used as potential anti-inflammatory agents in AD [84]. Starting from data reported in the literature that showed the ability of punicalagin to decrease NFAT activity in vitro [96] and considering that both punicalagin and EA after oral ingestion are available in plasma, the researchers studied if the PE and its mentioned isolated components had any inhibitory activity in vitro on NFAT or in vivo on the amyloid precursor protein/presenilin 1 (APP/PS1) transgenic AD mouse model [84].

PE was prepared using Wonderful pomegranates. The authors did not attribute the observed results to any particular component of PE, but in agreement with the literature, they found a relevant anti-inflammatory effect in the brains of AD mouse models treated with PE [84]. Even if the anti-inflammatory action of dietary PE has been shown in several different systems, the precise mechanism or bioactive component responsible for this effect is unclear.

The APP/PS1 line administered with PE were characterized by a remarkable behavioral improvement that could be related to the decrease in microgliosis, NFAT activity, and TNF-α concentration. A slight but significant decrease in Aβ plaques was found in mice fed with PE and this may contribute to the behavioral improvement observed. Nevertheless, in this work, the authors were not able to quantify the activities of β or γ-secretases or to measure the ratio between Aβ production and clearance [84]. Interestingly, in vitro data reported that PE, punicalagin, and EA decreased NFAT activity and cytokine secretion, while in mice fed with PE only, TNF-α secretion was decreased both in the brain and spleen, Table 1. Concluding, the idea was that pomegranate acting as a brain anti-inflammatory agent may contribute to reduce AD progression [84].

Epidemiological studies indicate that the use of non-steroidal anti-inflammatory drugs (NSAIDs) can reduce or retard the development of AD, in contrast to clinical trials where AD patients did not show any benefit when treated with NSAIDs. An interesting work has been recently published about this apparent discrepancy [97].

Due to the multifunctional nature of the bioactive substances present in the PF, in a study, it has been evaluated if dietary supplementation with pomegranate, and other fruits (figs and dates), can have any effect on inflammatory cytokines and ATP levels in an aged AD-like Aβ mice model (APP_sw_/Tg2576) compared to wild-type [85]. In accordance with previously published results [82], Tg2576 mice fed for 15 months with Oman pomegranate, figs, and dates showed decreased levels of Aβ_1–40_ and Aβ_1–42_ in the brain (cortex and hippocampus) with respect to the control mice. Furthermore, in the cerebrospinal fluid or plasma of AD patients, the levels of proinflammatory cytokines, especially IL-1β, IL-6, and TNF-α, increased in APP_sw_/Tg2576 mice. Interestingly, if APP_sw_/Tg2576 mice were fed with diet supplemented with pomegranate, figs, or dates, the levels of IL-1β, IL-6, and TNF-α decreased, Table 1 [85]. The result obtained was in line with other works present in the literature that highlighted the potential role of pomegranate that due to the high amount of polyphenols possesses a strong antioxidant activity able to protect the Tg2576 AD mice from the inflammation characteristic of AD [59,98,99,100].

In another study, the effect of PE against the OS was evaluated, in parallel in vitro, using cells treated with H_2_O_2_ and in vivo in mice with OS-induced Alzheimer’s symptoms. The experiments suggested that PE acted as a good antioxidant agent both in vitro and in vivo. Moreover, the results obtained in the mice indicated that PE inhibited neuronal cell death caused by Aβ-induced OS [86], Table 1.

As previous mentioned, there is a strict connection between AD and OS. The production of ROS due to Aβ can favor the structural damage of the cell membranes through lipidic peroxidation (LPO) and protein carbonyl formation that provokes oxidative neuronal cell death and cognitive decline in patients affected by AD [101,102]. Inspired by the promising results reported in the literature, some authors studied the effect of long-term dietary (15 months) supplementation of pomegranate on OS status in APPsw/Tg2576 mice [87]. When ROS damages lipids and proteins, there is production of MDA and protein carbonyl; thus, by measuring them it is possible to indirectly quantify the OS. It has been observed that in Tg2575 mice, there is a high production of MDA and protein carbonylation in the cerebral cortex and hippocampus, suggesting that the OS relate to AD. When the diet of AD mice was enriched by PJ, the MDA and protein carbonyl levels were strongly reduced showing that pomegranate acts as a direct antioxidant agent [87,103]. Moreover, in Tg2575 mice fed with pomegranate, the levels of different antioxidant enzymes such as glutathione (GSH), glutathione peroxidase (GPx), glutathione S transferase (GST), and superoxide dismutase (SOD) were maintained compared to wild controls, Table 1. These results suggest that Oman pomegranate in AD mice exhibits an antioxidant effect, probably due to the high levels of the polyphenols, that can contribute to ameliorate AD side effects by contrasting the OS. Finally, the mice treated with a long-term pomegranate supplementation diet showed a reduced activity of AChE and this is in agreement with data reported in the literature [87,104].

Recently, in an in vivo study in APPsw/Tg2576 mice, it was investigated if the dietary supplementation of 4% PE (Oman) could have a beneficial effect in preventing the loss of synaptic plasticity and neuroinflammation [88]. It is known that, in the brain of AD mice models, the expression of synaptic structural proteins is decreased compared to control mice, a first step towards neuronal loss [105,106,107,108]. When APPsw/Tg2576 mice were fed with 4% PE for 15 months, the expression of synaptic proteins named PSD-95, Munc 18-1, SNAP25, synaptophysin, and the ratio of p-CaMKIIα/CaMKIIα (calcium/calmodulin-dependent protein kinase II) and pCREB/CREB (cyclic AMP–response-element-binding protein) were drastically increased compared to APPsw/Tg2576 mice nourished with a standard diet. Another protein involved in synaptic plasticity is mTOR (mechanistic target of rapamycin); alteration of mTOR signaling has been related to cognitive and behavioral deficits [109,110,111]. The 15-month-old APPsw/Tg2576 mice treated with 4% pomegranate diet showed activation of the PI3K–Akt–mTOR pathway suggesting that pomegranate may improve synaptic function also through this axis. All the results reported showed that a diet rich in pomegranate may contribute to reduce deficits in memory and cognition by increasing the synaptic plasticity.

As previously mentioned, dietary pomegranate supplementation can contribute to reduce chronic OS in AD mice, decreasing AChE activity and the Aβ_1–40_ and Aβ_1–42_ levels [79,82,87]. BACE1 cleaves the full length of APP to form the smaller soluble ectodomain fragment (sAPPβ) and the β-CTF [112]. In Tg2576 mice, high levels of BACE1, sAPPβ, and β-CTF were found compared to wild-type mice. However, when AD mice were fed with PE for 15 months, the expression of BACE1, sAPPβ, and β-CTF appeared deeply reduced, supporting the theory that pomegranate consumption inhibits BACE1 activity in AD.

In addition, another important aspect in AD pathogenesis is the inflammation that is deleterious to neurons but necessary to contribute to the clearance of Aβ deposits. In this study, AD mice treated with PE were more able to combat neuroinflammation by reducing the expression of TNF-α, IL-1β, inducible nitric oxide synthase (iNOS), chemokine C-C motif ligand 2 (CCL2), and IL-10β, when compared to APPsw/Tg2576 mice receiving a standard diet [88], Table 1. Moreover, in APPsw/Tg2576 mice fed with 4% PE, the expression of the protein Beclin-1 (bcl1) and Lipidated LC-3 (LC-3 type II) increased, indicating autophagy activity essential for removing Aβ aggregates [88], Table 1. In summary, the authors suggested that long-term supplementation with pomegranates can reduce inflammation, ameliorate synaptic plasticity, and alter APP processing.

## 3. Biotransformation of Pomegranate and Bioactivity of Its Metabolites in AD

Natural bioactive compounds possess an intrinsic multitarget nature showing at the same time anti-inflammatory, antioxidant, free-radical scavenging and metal chelation properties, cell signaling modulation, and anti-amyloidogenic activity [38,113].

Nevertheless, a common remark regarding dietary intake of natural compounds is whether they persist in the systemic circulation in physiologically relevant concentrations to perform their biological effects. Many phenolic metabolites reached a high plasma concentration (5–20 μM), while their parent compounds were undetected [114]. Information about the bioavailability of phenolic compounds has been researched by measuring their concentrations in plasma and urine after the ingestion of pure derivatives or of foodstuffs with a known content of the molecule of interest. Interestingly, 75 to 99% of the polyphenols ingested were not detected in the urine. This suggests that the phenolic compounds have either not been absorbed through the gut barrier, that they were absorbed and excreted in the bile, or they were metabolized by the colonic microflora or tissues. In fact, the plasma concentration of the flavonoids ingested rapidly decreased (elimination half-life period of 1–2 h) because they were absorbed in the small intestine. The rapid excretion is driven by the conjugation of the aglycone to sulfate and glucuronide groups. Studies report that the majority of phenolic compounds found in the plasma are unknown metabolites and not those ingested [115]. Understanding the precise mechanism of polyphenols absorption through the gastrointestinal tract is complex because several parameters need to be taken into account, including anatomical and physiological features, as well as the physicochemical properties of the chemical under study [116,117].

Several studies report that despite the high concentration of ellagitannins present in pomegranate, these polyphenols are not detectable in body fluids because they are easily hydrolyzed in EA [118]. As described in the previous paragraphs, EA as well as punicalagin have been largely studied for their potent antioxidant and anti-inflammatory properties, as well as for their ability to act as β-secretase inhibitors [66]. EA is considered a molecule with potent neuroprotective effects because alongside the mentioned properties, it is also a good iron chelator, an activator of different cell signaling pathways, and it is able to mitigate mitochondrial dysfunction [119].

**Figure 4:**
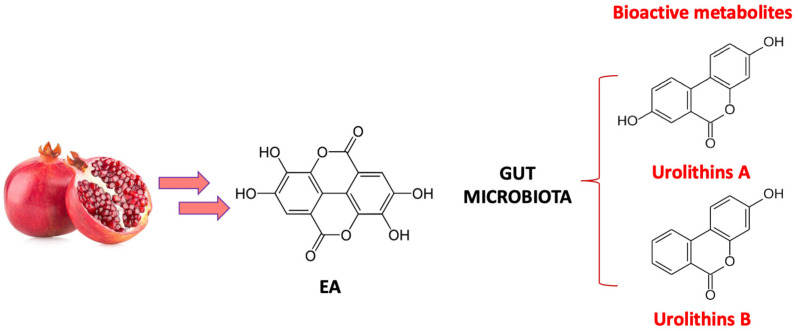
Biotransformation of EA by the gut microbiota in bioactive metabolites urolithins A and B.

Therefore, when PF, PJ, or PE were ingested, a high amount of polyphenols enter the body and they are rapidly metabolized into EA. Finally, the biotransformation of EA by the gut microbiota leads to urolithins metabolites (urolithins A and B, Figure 4), the major compounds found in human fluids and tissues [120].

In this context, it is important to mention the gut–microbiota–brain axis that refers to the interconnection between the gut bacteria and the brain. The gut–microbiota–brain axis is fundamental for maintaining homeostasis of the gastrointestinal, central nervous, and microbial systems of humans. The communication between the microbiota and the brain occurs through the vagus nerve, and numerous studies conducted on animal models suggest that the gut microbiota can have an effect on neurological disorders [121].

Urolithins are the major bioactive metabolites of pomegranate and they have largely been studied in several pathologies including neurodegenerative ones due to their potential ability to cross the blood–brain barrier (BBB) [52,122,123,124,125].

Based on the positive effect that dietary supplementation with pomegranates showed in different animal models and considering that the major metabolites found were urolithins, DaSilva et al. investigated if urolithins A and B and their methylated derivatives were able to reduce the neuroinflammation in murine BV-2 microglia and in human SH-SY5Y neurons. Moreover, they performed an in vivo study on AD (R1.40) mice treated with PE in order to investigate the effect on inflammatory biomarkers. An in vitro study performed using an (lipopolysaccharides) LPS-stimulated murine BV-2 microglia cell model suggested that urolithins can avoid neuronal cell death by inhibiting the production of NOS and the proinflammatory cytokines such as IL-6, TNF-α, and prostaglandin E_2_ (PGE_2_) [126]. Urolithins were able to reduce the acute OS induced by H_2_O_2_ in microglia and neurons, preserving the cell viability and decreasing the activity of caspase 9 and 3/7. Starting from these results, it has been hypothesized that urolithins can act as neuroprotective agents both in preventing inflammation and OS-mediated apoptosis by inhibiting caspase activation [126].

In another study, the neuroprotective activity of the metabolite urolithin A was evaluated towards mitochondrial dysfunction in a cellular model of early AD (SH-SY5Y-APP695 cells). The results showed that urolithin A did not have any effect on autophagy in SH-SY5Y-APP695 cells (Aβ_1-40_ level was unchanged with respect to control) and its action on mitochondrial function was limited. Interestingly, the data suggest that in the AD cell model treated with urolithin A, there were hormetic effects able to stimulate the transcription of several genes related to mitochondrial biogenesis [127].

Recently, a study has investigated the neuroprotective effect of urolithin B on aging-associated cognitive deficiency and brain injury. The antioxidant profile of urolithin B was characterized both in in vitro tests, such as DPPH, ABTS^+^, O_2_^.^, and ^.^OH, and in neuronal cells treated with H_2_O_2_ to provoke OS and apoptosis. Then, urolithin B was evaluated in an in vivo animal model of brain aging where D-galactose (D-gal) was administered by subcutaneous injection (150 mg/kg/d for 8 weeks) to induce AD-like symptoms. The results showed that urolithin B possessed an efficacious antioxidant profile in a free-radical-based assay and that it was able to inhibit cell apoptosis induced by H_2_O_2_ in cells. Moreover, in D-gal-treated aging mice, pretreatment with urolithin B showed a protective action against oxidative injury in the brain, suggesting that urolithin B promotes neuronal survival by protecting against OS and ultimately leading to an amelioration of neurological deficits and cognitive performance.

Starting from the data reported in the literature, it can be hypothesized that urolithins may be considered effective supplements to inhibit the OS related to memory impairment and brain injury, and neuroinflammation typical of neurological disorders.

## 4. Conclusions

Across the years, several efforts have been undertaken to understand the mechanism by which natural compounds can contribute to inhibit AD progression. In this review, we reported the in vitro and in vivo studies performed on PF, PJ, and PE in order to summarize the information, available at present in the literature, about the bioactive compounds and the main metabolites present in pomegranate.

Several in vivo studies showed that AD animal models fed with pomegranate are characterized by a reduction in reactive oxygen species formation, reduction of Aβ deposition thought BACE-1 inhibition, reduction in levels of hyperphosphorylation of the tau protein, and reduction of microglial activation. Moreover, pomegranate administration simultaneously induces maintenance of neuronal synaptic plasticity and acts as an anti-inflammatory agent. Certainly, further investigation needs to be conducted in human subjects; however, in vitro and in vivo animal experimental evidence suggests that pomegranate possesses an intrinsic multifunctional nature which is effective against AD symptoms.

Considering that the major bioactive pomegranate metabolites are EA and urolithins, their structure can inspire researchers to design new compounds that maintain the multifunctional characteristics of the natural ones and, at the same time, overcome the limitations of bioavailability related to nutraceuticals present in pomegranates.

## Figures and Tables

**Table 1:** Pomegranate’s beneficial action in AD animal models.

Animal Model	Diet Supplementation	Effects	Reference
APP_sw_/Tg2576	Pomegranate juice	↓ Aβ deposit ↑ cognitive function	[79]
APP_sw_/Tg2576	Oman pomegranate	↓ memory deficit ↓anxiety ↑ motor coordination	[82]
R1.40	Pomegranate extract	↓ Aβ deposit	[83]
APP/PS1	Pomegranate extract	↓ Aβ-stimulated ↓ TNF-α	[84]
APP_sw_/Tg2576	Pomegranate	↓ TNF-α ↓ IL-1β ↓ IL-6	[85]
ICR mice Injected with Aβ_1-42_	Pomegranate extract	↓ neuronal cell death	[86]
APPsw/Tg2576	Pomegranate juice (Oman)	↓ MDA ↓ protein carbonyl ↓ AChE ↑ SOD ↑GPx ↑GSH ↑ GST	[87]
APPsw/Tg2576	Pomegranate extract	↑ synaptic proteins ↓TNF-α ↓IL-1β ↓IL-10β ↓iNOS ↓CCL2 ↓bcl1 ↓LC-3	[88]

## Data Availability

Data sharing not applicable.
